# Association between Self-Reported Eating Rate, Energy Intake, and Cardiovascular Risk Factors in a Multi-Ethnic Asian Population

**DOI:** 10.3390/nu12041080

**Published:** 2020-04-13

**Authors:** Pey Sze Teo, Rob M. van Dam, Clare Whitton, Linda Wei Lin Tan, Ciarán G. Forde

**Affiliations:** 1 Clinical Nutrition Research Centre (CNRC), Singapore Institute of Food and Biotechnology Innovation (SIFBI), Agency for Science, Technology and Research (A*STAR), Singapore 117599, Singapore; Teo_Pey_Sze@sifbi.a-star.edu.sg; 2 Saw Swee Hock School of Public Health, National University of Singapore, Singapore 117549, Singapore; rob.van.dam@nus.edu.sg (R.M.v.D.); clarewhitton@nus.edu.sg (C.W.); linda_tan@nus.edu.sg (L.W.L.T.); 3 Department of Medicine, Yong Loo Lin School of Medicine, National University of Singapore, Singapore 119228, Singapore; 4 Department of Nutrition and Department of Epidemiology, Harvard T.H. Chan School of Public Health, Boston, MA 02115, USA; 5 Department of Physiology, Yong Loo Lin School of Medicine, National University of Singapore, Singapore 117593, Singapore

**Keywords:** self-reported eating rate, energy intake, obesity, cardiovascular, multi-ethnic, Asia

## Abstract

Eating faster is associated with greater body mass index (BMI), but less is known about the relationships between eating rate, energy intake, body composition, and cardio-metabolic risk factors in different Asian ethnic groups. Using data from the Singapore Multi-Ethnic Cohort (*n* = 7011; 21–75 y), we investigated associations between self-reported eating rate (SRER), with energy intake, body composition, blood pressure, and blood lipids. SRER and lifestyle was assessed using interviewer-administered questionnaires. Multivariable models were used to examine the associations of SRER with energy intake, body composition, blood pressure, and blood lipids after adjusting for covariates. General and abdominal overweight were defined as BMI ≥ 23 kg/m^2^ and waist circumference >90 cm (men) and > 80 cm (women), respectively. On average, faster eaters (vs. slower eaters) consumed 105 kcal/day more (*p* = 0.034), had ~5 kg higher body weight (*p* < 0.001), 1.3 kg/m^2^ higher BMI (*p* < 0.001), and 3.1 cm larger waist-circumference (*p* < 0.001). Faster eaters had significantly higher blood pressure, circulating triglycerides, and total-to-high-density lipoprotein cholesterol ratio than slower eaters. Faster eaters were twice as likely to develop general (multivariable-OR: 2.2; 95% CI, 1.8–2.6; *p* < 0.001), and abdominal (OR: 1.8; 95% CI, 1.5–2.2; *p* < 0.001) overweight than slower eaters. This association was observed across all subgroups by age, sex, and ethnicity. Findings suggest that SRER is a robust behavioral marker for increased risk of higher energy intake, obesity, and poor cardio-metabolic health, and a modifiable behavioral risk-factor for obesity prevention.

## 1. Introduction

Obesity is a major public health challenge and affects around 650 million adults worldwide [1]. While obesity has traditionally afflicted developed nations, low- and middle-income countries are also experiencing exponential increases in obesity rates. Having obesity is associated with a range of metabolic disorders responsible for much of the non-communicable diseases that are major causes of morbidity and mortality worldwide [2]. Identifying the dietary and behavioral drivers of excess energy intake can create opportunities for novel interventions to prevent obesity.

Obesity results from a sustained excess energy intake and the availability of inexpensive, energy-dense, and highly palatable foods appears to be a major driver of increases in obesity prevalence [3]. Public health interventions to prevent obesity include promoting healthy eating and re-shaping the food environment [4], with proposed limits on portion size and energy density and taxes to reduce the consumption of sugar-sweetened beverages [5]. Altering eating behavior by reducing the eating rate has become a hallmark of many weight control programs [6,7] and eating slowly may be a simple, cost-effective strategy to better regulate food intake and body weight. The rate of eating (g/min or kcal/min) acts as an indicator of appetite avidity and satiety sensitivity [8] and is a product of an individual’s drive to eat, and the textures within the food environment they choose to consume [9]. Eating rate is a stable behavior over time [10], in which the eating rate at an individual level is consistent from meal to meal [11,12] but can vary significantly between individuals [13]. Self-reported eating rate is often reported in large-scale epidemiological studies and enables classification groups of into slower, medium, or faster eating rates at a population level [14]. Evidence from epidemiological studies has consistently demonstrated that individuals that self-report eating at a faster rate tend to consume more energy [15] and are more likely to be overweight [15,16,17]. Prospective studies have also indicated an association between a self-reported faster eating rate and greater weight gain and a higher risk of obesity over longer periods [18,19]. Individuals that self-report eating faster have also been shown to have a higher risk of type-2-diabetes [20], the metabolic syndrome [21], and non-alcoholic fatty liver disease [22]. Therefore, self-reported eating rate (SRER) could potentially be a robust behavioral marker to identify obesity risk and support obesity prevention strategies.

However, gaps remain in the existing literature. First, previous population-based research mainly focused on the association between SRER, body weight, and BMI, but limited information pertaining to energy intake as a potential mediator is available. Second, few studies with large sample sizes have been conducted to elucidate the association between SRER and cardio-metabolic health. In addition, most studies were limited to East-Asian (predominantly Japanese) and Western populations, and to our knowledge, no study to date has explored these associations in a multi-ethnic Southeast Asian population. Singapore has a multi-ethnic population consisting of three major Asian ethnic groups, Chinese, Malays, and Indians, that represent a large part of the world population with substantially different cuisines than Japanese and Western populations. It is currently unclear whether cultural differences in food preferences and eating behaviors influence relationships between SRER, energy intake, obesity, and associated metabolic disorders differently across different ethnic groups. 

The current study examined the association between self-reported eating rate, energy intake, body composition, blood pressure, and blood lipids in a population-based multi-ethnic cohort in Singapore. A secondary objective was to examine whether the association between SRER, body composition, and cardio-metabolic health differs by age, sex, and ethnicity.

## 2. Materials and Methods 

### 2.1. Study Population

The Singapore Multi-Ethnic Cohort Phase 2 study (MEC2) is a population-based cohort study of Singaporean citizens and permanent residents aged 21–75 years including the three major ethnic groups: Chinese, Indian, and Malay. This study uses the data collected from the follow-up of the MEC2 (http://blog.nus.edu.sg/sphs/the-first-sphs-follow-up/). Detailed information on MEC2 and its follow-up is available at http://blog.nus.edu.sg/sphs/ and a cohort profile described by Tan et al. [23]. Data collection of the MEC2 follow-up consists of two parts, a home interview and a physical examination at the health screening center. The present study included those who participated in both interview and health screening sessions (*n* = 7314), and excluded those with missing data (*n* = 86), invalid energy intake (i.e., had extreme energy intake of ≤500 kcal/day or ≥6000 kcal/day) (*n* = 139), and suffering from major chronic diseases (*n* = 78), i.e., cancer, heart attack, and stroke. A final sample of 7011 participants were included in the current analysis. Informed consent was obtained from all participants prior to the study, and the study protocol was approved by the Institutional Review Board of the National University of Singapore (NUS-IRB-B-16-125).

### 2.2. Assessment of Self-Reported Eating Rate, Diet and Covariates

Trained staff performed face-to-face home interviews to collect detailed information from all participants. Data were collected on sociodemographic characteristics, medication use, personal and family medical history, and lifestyle factors. The rate of eating was assessed using a previously published approach [14], by the response to the following question: “How fast is your rate of eating?”, chosen from five qualitative categories, namely “very slow”, “relatively slow”, “medium”, “relatively fast”, and “very fast”. Dietary intakes were assessed using a validated semi-quantitative food frequency questionnaire (FFQ) [24] that included 163 items, with additional sub-questions on food sub-types and cooking methods. Participants were asked to consider their intake over the past year when answering. A visual aid was also used to help quantify the standard portion sizes with the assistance of trained interviewers. Physical activity was assessed using the Singapore Prospective Study Program Physical Activity Questionnaire (SP2PAQ), a previously validated physical activity questionnaire, which covers the duration and frequency of transportation, leisure, and household activities [25]. The metabolic equivalent task units (METs), the amount of energy expended while at rest, were calculated for each activity type and duration using the Ainsworth et al. compendium [26]. 

### 2.3. Assessment of Body Composition, Blood Pressure, and Lipid Profiles

Participants subsequently attended a health screening where anthropometric measurements and blood pressure were taken, and blood samples collected. All anthropometric measurements were assessed according to WHO standards. Height and body weight were measured to the nearest 0.1 cm and 0.1 kg, respectively, by trained personnel. Participants were instructed to remove their shoes before having their height measured using a portable stadiometer (200 series; SECA, Hamburg, Germany), with the head positioned in the Frankfurt plane. They were also instructed to remove heavy belongings from their clothing before their body weight was measured using a digital scale (700 series; SECA) [23]. Body mass index (BMI) was calculated by dividing weight (kg) by height squared (m^2^). In this study, the Asian cut-off of 23 kg/m^2^ was used to identify individuals at moderate to high risk of obesity-related diseases [27,28], as Asians often have more body fat for a given BMI as compared with individuals of European origin [28]. Waist circumference (WC) was measured to the nearest 0.1 cm using a tape measure positioned at the mid-point between the last rib and the iliac crest. The WC Asian cut-offs, i.e., > 80 cm for women and > 90 cm for men, were used for abdominal obesity [29]. Systolic and diastolic blood pressure was measured twice with a 5-min interval, using an automated blood pressure monitor (Dinamap Carescape V100, General Electric Company, Helsinki, Finland). However, if measurements differed by more than 10 (systolic) or 5 mmHg (diastolic) between the first two readings, a third measurement was taken and the mean of the two closest measurements would be derived. During the health screening visit, the random or overnight-fasting venous blood samples were also drawn from participants to analyze the concentrations of serum total cholesterol, high-density lipoprotein cholesterol (HDL-C), low-density lipoprotein cholesterol (LDL-C), and triglycerides (TGs). The total-to-HDL-cholesterol ratio was calculated by dividing the total cholesterol level with the level of HDL-C. All collected blood samples were analyzed using an automated chemistry analyzer (DxC 600, Beckman Coulter, Brea, CA, USA).

### 2.4. Statistical Analyses

Based on the distribution of the self-reported eating rate (SRER) data, we collapsed the lowest two (“very slow” and “relatively slow”) and the highest two (“relatively fast” and “very fast”) SRER categories into two groups “slow” and “fast”, respectively. Descriptive statistics were reported as mean ± SD, unless otherwise indicated. All variables were tested for normality using the Kolmogorov–Smirnov test prior to any statistical comparison. Analysis of covariance (ANOVA) was used to determine the differences of all continuous variables across the eating speed groups, while the Person Chi-Square test was used to evaluate the differences of the categorical variables across the SRER groups. Differences in the prevalence of overweight (BMI > 23 kg/m^2^) and abdominal overweight (WC > 80 cm for women; > 90 cm for men) among eating speed groups were stratified by sex, age, and ethnicity and evaluated using Chi-square tests for trend. Multivariable ANOVA was used to determine the influence of different SRER groups on the dietary energy intake, body composition outcomes, blood pressure, and blood lipid profiles, by adjusting all known potential confounders including age (years), sex (male or female), ethnicity (Chinese, Malay, Indian), education level (primary or below, secondary, higher education including vocational, university), total physical activity (MET-min/week), smoking (yes or no), and alcohol consumption status (yes or no). For outcome variables of blood pressure and blood lipid profiles, BMI was also further included in those adjusted multivariable ANOVA models. In addition, known hypertensives (*n* = 1143) and statin users (*n* = 1421) were excluded from the respective multivariable ANOVA analyses, where blood pressure and blood lipids were the outcome variables, to avoid confounding. Multivariable logistic regression model analysis was also performed to examine the odd ratios of general and abdominal overweight as assessed by BMI and WC across different SRER groups, with adjustment for all the potential confounders. The interactions between self-reported eating rate with sex, age, and ethnicity were assessed by adding multiplicative interaction terms to the fully adjusted multivariable model. All statistical analyses were performed using the IBM SPSS for Window version 23.0 (IBM, Armonk, NY, USA) and *p*–values < 0.05 were considered as statically significant.

## 3. Results

Table 1 shows socio-demographic, dietary, and lifestyle characteristics according to self-reported eating rate (SRER). Of the 7011 participants, Chinese was the largest ethnic group (71.1%), followed by Indians (14.7%), Malays (9.0%), and others (5.2%), which is generally in line with population ethnic distribution for Singapore. The mean age of participants was 49.8 ± 13.0 years, and 44.8% were men. The younger age was associated with a faster eating rate (*p* < 0.001); whereas the daily dietary energy intake increased significantly across the SRER groups (*p* < 0.001), i.e., slow, medium, and fast. 

The average BMI was 24.9 ± 4.6 kgm^2^ and the majority (62.1%) were overweight according to Asian criteria (Table 2). According to the unadjusted analysis, participants reporting a faster SRER had significantly higher height, body weight, BMI, waist circumference (WC), systolic and diastolic blood pressure, levels of triglycerides (TGs), and total-to-HDL-cholesterol ratio (cholesterol/HDL ratio) than those reporting lower SRER. However, participants in the “fast” SRER group reported a lower systolic blood pressure than their counterparts in the “medium” group. In addition, faster SRER was associated with lower levels of high-density lipoprotein cholesterol (HDL–C) (*p* < 0.001).

**Figure 1:**
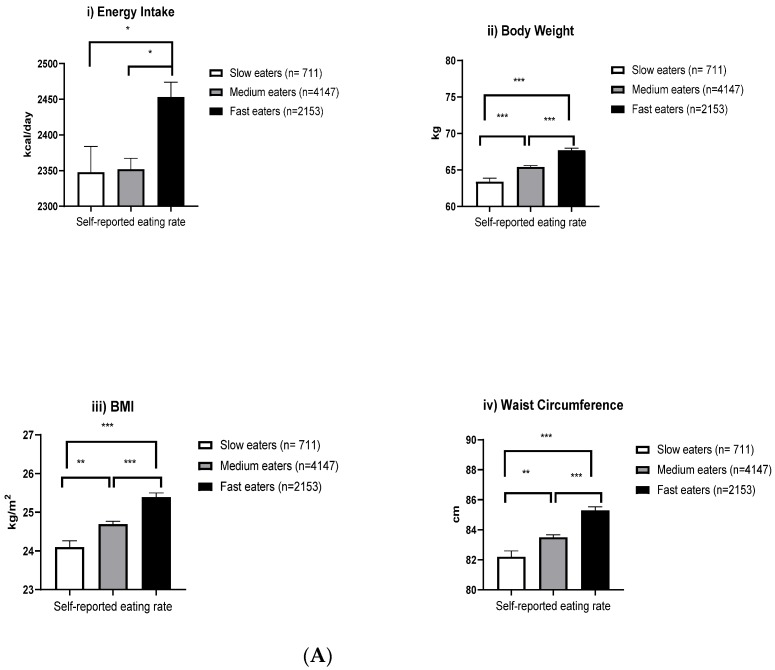

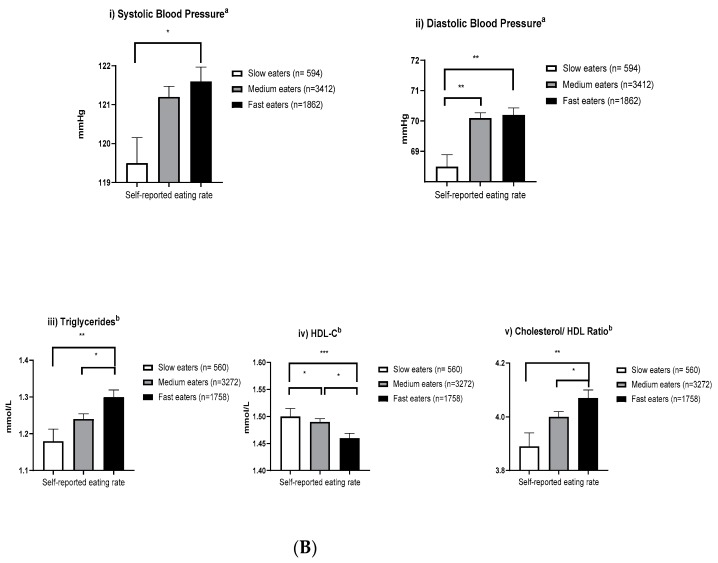
Multivariable ANOVA analyses of the association between self-reported eating rates, and (**A**) Energy intake, and body composition profiles, (**B**) Blood pressure ^a^, and blood metabolic ^b^ profiles in the Singapore Multi-Ethnic Cohort 2, *n* = 7011, after adjusting by age (years), sex, ethnicity, education level, total physical activity (MET-min/week), smoking, and alcohol drinking status. Participants with ^a^ diagnosed hypertension, *n* = 1143; ^b^ taking statins, *n* = 1421 were excluded from the analysis models. Significant different at * *p* < 0.05, ** *p* < 0.01, *** *p* < 0.001.

We subsequently examined SRER in relation to energy intake, body composition, blood pressure, and metabolic profiles in multivariable models with adjustment for age, sex, ethnicity, education level, total physical activity, smoking, and alcohol consumption status (Figure 1A,B). Participants in the “fast” SRER group had a higher daily energy intake (105 kcal/day, *p* = 0.034), higher body weight (5 kg, *p* < 0.001), higher BMI (1.3 kg/m^2^, *p* < 0.001), larger WC (3.1 cm, *p* < 0.001), higher systolic (2.1 mmHg, *p* = 0.018) and diastolic (1.7 mmHg, *p* < 0.001) blood pressure, higher concentrations of TG (0.1 mmol/l, *p* = 0.007), and a higher total-to-HDL-cholesterol ratio (0.2, *p* = 0.001), compared with those in the “slow” SRER group, after multivariable adjustment. Participants with “fast” SRER also had significantly lower concentrations of HDL-C (−0.04 mmol/l, *p* < 0.001) than those with “slow” SRER. Additional adjustment for dietary energy intake did not substantially attenuate the associations between SRER and body composition and the association remained significant. However, the associations of SRER with systolic blood pressure and lipids became substantially weaker and non-significant after further adjustment for BMI, suggesting that the association between SRER and cardiovascular risk factors was largely mediated by adiposity. Only the association of faster SRER with higher diastolic blood pressure remained statistically significant (*p* = 0.02).

Table 3 shows multivariable adjusted odd ratios (ORs) for associations between SRER and overweight by sex, ethnic, and age. Eating “fast” (vs. “slow”) was directly associated with both general (OR 2.2; 95% CI, 1.8–2.6; *p* < 0.001) and abdominal (OR 1.8; 95% CI, 1.5–2.2; *p* < 0.001) overweight. Similar associations were also found between eating “medium” (vs. “slow”) and both general (OR 1.5; 95% CI, 1.3–1.8; *p* < 0.001) and abdominal (OR 1.4; 95% CI, 1.2–1.7; *p* < 0.001) overweight. These associations of SRER with general and abdominal overweight were consistently observed across both sexes, the three main ethnic groups (Chinese, Malays, and Indians), and different age groups. There were no significant interactions between age group, ethnicity, and sex with SRER on overweight. However, of the ethnic groups, Malays classified as “fast” eating (vs. “slow”) tended to have the highest odd ratio of being overweight, whereas the older adults aged 55 years and above with “fast” eating tended to have the lowest odd ratio of being overweight (Table 3).

## 4. Discussion

In this large population-based study, we demonstrated a significant association between faster SRER, energy intake, body composition, and biological markers of cardiovascular disease across three major Asian ethnic groups, Chinese, Malays, and Indians. Faster eaters consumed on average 105 kcal more per day, were approximately 5 kg heavier, had a 1.3 kg/m^2^ higher BMI, and a 3.1 cm larger waist circumference, after multivariable adjustment. In addition, those self-reporting as faster eaters also had significantly worse cardiovascular risk factors, including higher blood pressure, circulating TG, and total-to-HDL-cholesterol ratio, compared to those classified as slower eaters. Identifying as a faster eater may provide new insights on food-related behaviors that promote greater energy intake and higher body weight. For example, faster eaters were twice as likely to be overweight than slower eaters, with consistent associations across sex, age, and ethnic groups. Having a higher self-reported eating rate may thus represent a robust behavioral marker that is consistently associated with weight status and cardiovascular health. To the best of our knowledge, the current study is the first to demonstrate these associations in a large Southeast Asia population of mixed ethnicity.

Individuals with a faster SRER had a significant increase in daily energy intake, body weight, and a greater likelihood of being overweight compared with their slower counterparts. These results are consistent with previous cross-sectional [15,16,17,30,31] and longitudinal studies [18,19] in both East Asian and Western populations that have reported direct associations between eating rate, energy intake, and obesity. The current adjusted odds ratios show that the likelihood of having overweight and obesity is 2.1 (95% CI, 1.8–2.6) times higher for faster eaters than slower eaters, and is highly compatible with the pooled odd ratio of 2.15 (95% CI, 1.84–2.51) in a recent meta-analysis from 23 published observational studies [32]. In line with these findings, a meta-analysis of experimental laboratory-based studies also found a significant increase in ad-libitum energy intake when participants ate at a faster rate [33]. These cross-over experimental behavioral studies on two to four separate days show that an increase in the within-meal eating rate of approximately 20% (g/min) results in an increase in ad-libitum energy intake of between 10% and 15% [34,35,36]. A proposed mechanism for this association is that rapid food intake results in shorter oro-sensory exposure time during consumption per kcal consumed, which may reduce the impact of satiety signals perceived during consumption [37,38,39], which in turn may reduce the opportunity to orally estimate the acute calorie intake within a meal. Previous research has shown that faster eating rates result in a slower onset of satiation and a weaker satiety response, in terms of both neuroendocrine signals and subjective feelings of satiety per kcal consumed [40,41,42]. This further supports the conclusion that eating faster is a robust behavioral marker of the maladaptive dietary behaviors that lead to sustained positive energy balance, weight gain, and increased obesity risk.

Notably, we also observed that faster eating was associated with higher systolic and diastolic blood pressure, higher circulating concentrations of TG, higher total-to-HDL-cholesterol ratio, and lower concentrations of HDL-cholesterol after adjusting for potential confounders. However, these associations were largely attenuated after additional adjustment of BMI. Only the association between SRER and diastolic blood pressure remained statistically significant. These results are in agreement with those of previous studies [31,43], indicating that the association between SRER and cardiovascular risk factors is mainly mediated by greater weight gain. From a public health standpoint, SRER may be a simple yet effective behavioral marker for an increased risk of weight gain and associated cardio-metabolic markers of health.

Across sex, ethnicity, and age subgroups, faster eaters were consistently more likely to be overweight than participants that were slower eaters. Of the three predominant ethnic groups in Singapore, Malays with faster eating rates tended to have higher multivariable-adjusted odds ratio of being overweight, i.e., 3 times more likely to be overweight when compared to slow eaters. Beyond differences in the rate of eating, such an ethnic difference may also reflect differences in physiology [44], dietary habits [45], and cultural food practices [46] between the different ethnic groups. Individuals adapt their eating rates based on the food form and texture that is being consumed [47]. Previous research has shown that the impact of an increased eating rate can be further increased when combined with the consumption of higher energy-dense foods, to promote higher energy intake rates (kcals/min) [48,49]. Future research should characterize the foods that promote greater energy intake rates in the Singaporean diet, and explore their impact on energy intake, body composition, and metabolic health in our population.

Findings from the current study are in line with previous evidence that supports a direct association between SRER and energy intake, body composition, and metabolic risk factors. SRER has previously been identified as a modifiable risk factor and intervention target for obesity management, with advice to ‘slow the rate of consumption to better control body weight’ [50]. Several approaches have been applied to reduce the eating rate using external cues, including providing digital feedback to modify the eating rate using electronic devices, or vibrio-tactile sensations to slow down the within-meal eating rate [51,52,53]. These approaches have yielded some success in reducing the energy intake and body weight in the short term. Nevertheless, there remain challenges with longer term adherence to a slower eating regime that relies on devices to cue eating behaviors during a meal. For example, women within a weight control program successfully reduced their eating rate by receiving advice to pause between bites and to cut food into smaller pieces, but the change in the eating rate were not maintained over time [7]. Alternatively, the eating rate can be modified through the use of food textures, which has been shown to produce a significant reduction in acute energy intake without the attendant loss of meal satisfaction or enjoyment [36]. Future research is needed to explore the efficacy of eating rate modification strategies that combine the individual and food-based approaches to modify the eating rate at a population level, and in the longer term.

A key strength of this present study was the use of a large multi-ethnic sample from the general population and the detailed information on potential confounders, including socio-demographic factors, dietary, and lifestyle practices. In addition, body weight, height, and WC were measured by trained staff, instead of self-reporting, which increases the accuracy of these measurements. However, the dietary intake and eating rate data were self-reported and therefore subject to measurement error. The residual confounding by unmeasured or imperfectly measured dietary or lifestyle factors may also still exist. The blood biochemical data analyzed from the random venous blood samples may not be comparable with the overnight-fasting collection and may be subject to underestimation. The current study reported differences in energy intake, body composition, and cardio-metabolic factors as they relate to differences in SRER. A recent review of the area highlights genetic, physiological, and environmental factors that are known to influence an individual’s eating rate [54]. Future studies should explore some of the reasons for differences in SRER, including potential lifestyle and environmental factors that may play a role. The cross-sectional nature of the study limits causal inference on the role of SRER on changes in body weight and adiposity and whether this is mediated by long-term differences in energy intake. Previous research has demonstrated a relationship between faster eating, greater energy intake, and prospective changes in body composition among a population of pre-school children [9]. Another study showed changes in body weight among a cohort of males over 8 years, where those reporting a higher SRER gained an average of 1.9 kg, compared to those with a lower SRER, who only gained 0.8 kg [19]. Future longitudinal interventions are required to investigate whether reducing SRER can have an impact on long-term energy intake and adiposity. As the eating rate is a product of both an individual’s drive to eat and the food environment they select to consume (i.e., texture, energy density) [54], future research should investigate the energy intake rate (kcal/min) of an individual’s diet, to establish whether the association between faster eating and body composition is caused by an individual’s drive to eat, or the energy intake rate of the foods that most contribute to greater energy intakes, or both. 

## 5. Conclusions

We showed that SRER was associated with higher energy intake, greater adiposity, and worse cardio-metabolic health. Significant direct associations were observed between SRER, energy intake, adiposity, and cardiovascular markers in a large multi-ethnic Southeast Asian population-based cohort. Fast eaters were twice as likely to be overweight than slow eaters and associations were consistent across age, sex, and three major Asian ethnic groups (Chinese, Indians, and Malays), which together comprise a large proportion of the world population. Unlike traditional dietary indices of non-communicable disease risk, SRER provides a robust behavioral marker that is consistently associated with increased energy intake and risk of obesity and poor cardio-metabolic health. Future research should use SRER to identify at-risk populations and develop tailored intervention approaches that slow down the rate of energy intake across different age and ethnic groups.

## Figures and Tables

**Table 1:** General characteristics of participants according to tertile groups of self-reported eating rate in the Singapore Multi-Ethnic Cohort 2, *n* = 7011.

	Self-Reported Eating Rate			*p* Value
	Slow (*n* = 711)	Medium (*n* = 4147)	Fast (*n* = 2153)	
	Mean ± SD			
Age, years	51.63 ± 14.38 ^a,^***	50.74 ± 12.72 ^a,^***	47.44 ± 12.62	<0.001
Sex, %				<0.001
Men	9.5	53.2	37.3	
Women	10.7	64.0	25.4	
Ethnic group, %				<0.001
Chinese	9.8	57.4	32.8	
Malay	11.7	71.0	17.3	
Indian	10.4	59.3	30.3	
Others	11.6	62.3	26.2	
Highest education attainment, %				<0.001
Primary or below	10.7	64.8	24.5	
Secondary	10.1	66.1	23.8	
Higher education inc. vocational	10.5	58.1	31.4	
University	9.3	49.5	41.2	
Smokers, %	9.4	56.2	34.4	0.004
Alcohol drinkers, %	9.8	47.3	42.8	<0.001
Dietary energy intake, kcal/day	2307.82 ± 995.69 ^a,^***	2308.39 ± 986.12 ^a,^***	2550.98 ± 1054.83	<0.001
Total physical activity, MET-min/week	1113.57 ± 1054.94 ^a,^*	1166.66 ± 1027.84	1219.70 ± 1048.57	0.035

Values are presented as mean ± SD unless otherwise noted. Significant different from ^a^ fast eaters at * *p* < 0.05, *** *p* < 0.001.

**Table 2:** Body composition, blood glucose, and lipid profiles, of participants according to tertile groups of self-reported eating rate in the Singapore Multi-Ethnic Cohort 2, *n* = 7011 ^1^.

	Total (*n* = 7011)	Self-Reported Eating Rate			*p*–Trend
		Slow (*n* = 711)	Medium (*n* = 4147)	Fast (*n* = 2153)	
	Mean ± SD				
Body weight, kg	65.90 ± 14.14	62.96 ± 14.06 ^a,^***^,b,^**	64.88 ± 13.66 ^a,^***	68.85 ± 14.61	<0.001
Height, m	1.63 ± 0.09	1.61 ± 0.09 ^a,^***	1.62 ± 0.09 ^a,^***	1.65 ± 0.09	<0.001
BMI, kg/m^2^	24.88 ± 4.64	24.14 ± 4.88 ^a,^***^,b,^**	24.78 ± 4.64 ^a,^***	25.30 ± 4.54	<0.001
WC, cm	83.93 ± 11.78	82.29 ± 12.63 ^a,^***	83.43 ± 11.59 ^a,^***	85.42 ± 11.69	<0.001
Systolic blood pressure, mmHg	124.32 ± 19.28	123.38 ± 19.64 ^b,^*	125.11 ± 19.78 ^a,^***	123.11 ± 18.09	<0.001
Diastolic blood pressure, mmHg	71.00 ± 10.70	69.32 ± 10.27 ^a,b,^***	70.99 ± 10.73 ^a,^*	71.58 ± 10.73	<0.001
Total cholesterol, mmol/L	5.54 ± 1.07	5.53 ± 1.08	5.56 ± 1.07	5.52 ± 1.06	0.425
LDL-C, mmol/L	3.54 ± 0.96	3.50 ± 1.00	3.55 ± 0.97	3.54 ± 0.93	0.418
HDL-C, mmol/L	1.48 ± 0.40	1.52 ± 0.41 ^b,^*^,a,^***	1.49 ± 0.40 ^a,^***	1.43 ± 0.39	<0.001
TG, mmol/L	1.31 ± 0.86	1.24 ± 0.80 ^a,^**	1.30 ± 0.84 ^a,^*	1.35 ± 0.92	0.005
Total-to-HDL-cholesterol ratio	3.99 ± 1.22	3.85 ± 1.13 ^b,^*^,a,^***	3.95 ± 1.19 ^a,^***	4.10 ± 1.28	<0.001

^1^ Unadjusted data. BMI, body mass index; WC, waist circumference; HC, hip circumference; WHR, Waist-Hip Ratio; LDL-C, low-density lipoprotein cholesterol; HDL-C, high-density lipoprotein cholesterol; TG, triglycerides. Values are presented as mean ± SD unless otherwise noted. Significant different from ^a^ fast; ^b^ medium eaters at * *p* < 0.05, ** *p* < 0.01, *** *p* < 0.001.

**Table 3:** Multivariable logistics models of odd ratios (ORs) for being overweight, assessed by BMI and waist circumference (WC) according to self-reported eating rate (SRER) in the Singapore Multi-Ethnic Cohort 2, by sex, ethnic, and age groups.

Variables	Overweight BMI > 23 kg/m^2^		Abdominal Overweight WC > 90 cm (Men); >80 cm (Women)	
	Odd Ratio ^a^ (95% CI)	Prevalence (%)	Odd Ratio ^a^ (95% CI)	Prevalence (%)
**Overall**				
Slow	1.00 (reference)	51.7	1.00 (reference)	35.8
Medium	1.54 (1.30, 1.83) ***	61.3	1.40 (1.18, 1.67) ***	43.0
Fast	2.17 (1.80, 2.60) ***	67.2	1.84 (1.52, 2.21) ***	44.1
**Subgroups**				
**Sex-Men**				
Slow	1.00 (reference)	58.4	1.00 (reference)	33.2
Medium	1.43 (1.10, 1.85) **	66.4	1.32 (1.01, 1.73) *	37.9
Fast	2.12 (1.61, 2.80) ***	73.4	1.85 (1.39, 2.45) ***	42.0
**Sex-Women**				
Slow	1.00 (reference)	47.0	1.00 (reference)	37.8
Medium	1.56 (1.24, 1.94) ***	57.8	1.43 (1.13, 1.78) **	46.3
Fast	2.02 (1.57, 2.59) ***	59.9	1.71 (1.33, 2.20) ***	46.4
**Ethnicity-Chinese**				
Slow	1.00 (reference)	43.0	1.00 (reference)	28.7
Medium	1.46 (1.20, 1.77) ***	52.1	1.27 (1.02, 1.57) *	33.7
Fast	2.09 (1.69, 2.58) ***	62.1	1.77 (1.41, 2.22) ***	38.1
**Ethnicity-Malay**				
Slow	1.00 (reference)	73.0	1.00 (reference)	48.7
Medium	1.69 (0.92, 2.93)	81.7	1.58 (0.94, 2.63)	61.0
Fast	3.08 (1.40, 6.79) **	88.1	2.11 (1.13, 3.95) *	62.4
**Ethnicity-Indian**				
Slow	1.00 (reference)	72.9	1.00 (reference)	54.2
Medium	1.65 (1.03, 2.67) *	81.2	2.0 (1.29, 3.07) **	66.5
Fast	1.77 (1.05, 2.99) *	82.1	2.0 (1.23, 3.13) **	64.5
**Age-20–34 years**				
Slow	1.00 (reference)	41.7	1.00 (reference)	21.7
Medium	1.54 (1.00, 2.37) *	53.7	1.46 (0.89, 2.40)	29.4
Fast	2.20 (1.40, 3.47) **	61.2	1.96 (1.17, 3.29) *	31.0
**Age-35–54 years**				
Slow	1.00 (reference)	51.5	1.00 (reference)	33.7
Medium	1.79 (1.36, 2.37) ***	65.7	1.54 (1.16, 2.06) **	44.4
Fast	2.41 (1.79, 3.23) ***	70.0	1.98 (1.45, 2.68) ***	45.5
**Age->55 years**				
Slow	1.00 (reference)	55.7	1.00 (reference)	42.9
Medium	1.23 (0.96, 1.58)	58.2	1.16 (0.91, 1.50)	45.3
Fast	1.77 (1.34, 2.35) ***	65.8	1.50 (1.13, 1.98) **	48.9

^a^ Adjusting for age (years), sex, ethnicity, highest education level, smoking status and alcohol drinking status, and total PA (METmin/week). All variables stratified by (1) sex were not adjusted for sex, (2) ethnic groups were not adjusted ethnicity, (3) age groups were not adjusted by age. Significant different at * *p* < 0.05, ** *p* < 0.01, *** *p* < 0.001.

## References

[1] World Health Organization Obesity and Overweight. Key facts. https://www.who.int/en/news-room/fact-sheets/detail/obesity-and-overweight.

[2] Hruby A., Manson J.E., Qi L., Malik V.S., Rimm E.B., Sun Q., Willett W.C., Hu F.B. (2016). Determinants and Consequences of Obesity. Am. J. Public Health.

[3] Romieu I., Dossus L., Barquera S., Blottière H.M., Franks P.W., Gunter M., Hwalla N., Hursting S.D., Leitzmann M., Margetts B. (2017). Energy balance and obesity: What are the main drivers?. Cancer Causes Control.

[4] Swinburn B., Caterson I., Seidell J., James W. (2004). Diet, nutrition and the prevention of excess weight gain and obesity. Public Health Nutr..

[5] Public Health England (2015). Sugar Reduction: The Evidence for Action.

[6] Brownell K.D. (2000). LEARN Program for Weight Management 2000.

[7] Spiegel T.A., Wadden T.A., Foster G.D. (1991). Objective measurement of eating rate during behavioral treatment of obesity. Behav. Ther..

[8] Llewellyn C.H., van Jaarsveld C.H., Boniface D., Carnell S., Wardle J. (2008). Eating rate is a heritable phenotype related to weight in children. Am. J. Clin. Nutr..

[9] Forde C.G., Fogel A., McCrickerd K. (2019). Children’s Eating Behaivors and Energy Intake: Overlapping Influences and Opportunities for Intervention. Nestle Nutr. Inst. Workshop Ser..

[10] Ioakimidis I., Zandian M., Eriksson-Marklund L., Bergh C., Grigoriadis A., Sodersten P. (2011). Description of chewing and food intake over the course of a meal. Physiol. Behav..

[11] Guy-Grand B., Lehnert V., Doassans M., Bellisle F. (1994). Type of Test-meal Affects Palatability and Eating Style in Humans. Appetite.

[12] McCrickerd K., Forde C.G. (2017). Consistency of Eating Rate, Oral Processing Behaviours and Energy Intake across Meals. Nutrients.

[13] Ketel E.C., Aguayo-Mendoza M.G., de Wijk R.A., de Graaf C., Piqueras-Fiszman B., Stieger M. (2019). Age, gender, ethnicity and eating capability influence oral processing behaviour of liquid, semi-solid and solid foods differently. Food Res. Int..

[14] Otsuka R., Tamakoshi K., Yatsuya H., Murata C., Sekiya A., Wada K., Zhang H.M., Matsushita K., Sugiura K., Takefuji S. (2006). Eating Fast Leads to Obesity: Findings Based on Self-administered Questionnaires among Middle-aged Japanese Men and Women. J. Epidemiol..

[15] van den Boer J.H.W., Kranendonk J., van de Wiel A., Feskens E.J.M., Geelen A., Mars M. (2017). Self-reported eating rate is associated with weight status in a Dutch population: A validation study and a cross-sectional study. Int. J. Behav. Nutr. Phys. Act..

[16] Maruyama K., Sato S., Ohira T., Maeda K., Noda H., Kubota Y., Nishimura S., Kitamura A., Kiyama M., Okada T. (2008). The joint impact on being overweight of self-reported behaviours of eating quickly and eating until full: Cross sectional survey. BMJ.

[17] Sasaki S., Katagiri A., Tsuji T., Shimoda T., Amano K. (2003). Self-reported rate of eating correlates with body mass index in 18-y-old Japanese women. Int. J. Obes..

[18] Gerace T.A., George V.A. (1996). Predictors of Weight Increases over 7 Years in Fire Fighters and Paramedics. J. Prev. Med..

[19] Tanihara S., Imatoh T., Miyazaki M., Babazono A., Momose Y., Baba M., Uryu Y., Une H. (2011). Retrospective longitudinal study on the relationship between 8-year weight change and current eating speed. Appetite.

[20] Mochizuki K., Misaki Y., Miyauchi R., Takabe S., Shimada M., Kuriki K., Ichikawa Y., Goda T. (2012). A higher rate of eating is associated with higher circulating interluekin-1β concentrations in Japanese men not being treated for metabolic diseases. Nutrition.

[21] Zhu B., Haruyama Y., Muto T., Yamazaki T. (2015). Association between eating speed and metabolic syndrome in a three-year population-based cohort study. J. Epidemiol..

[22] Lee S., Ko B.-J., Gong Y., Han K., Lee A., Han B.-D., Yoon Y.J., Park S., Kim J.-H., Mantzoros C.S. (2016). Self-reported eating speed in relation to non-alcoholic fatty liver disease in adults. Eur. J. Nutr..

[23] Tan K.H.X., Tan L.W.L., Sim X., Tai E.S., Lee J.J.-M., Chia K.S., van Dam R.M. (2018). Cohort Profile: The Singapore Multi-Ethnic Cohort (MEC) study. Int. J. Epidemiol..

[24] Whitton C., Ho J.C.Y., Tay Z., Rebello S.A., Lu Y., Ong C.N., van Dam R.M. (2017). Relative Validity and Reproducibility of a Food Frequency Questionnaire for Assessing Dietary Intakes in a Multi-Ethnic Asian Population Using 24-h Dietary Recalls and Biomarkers. Nutrients.

[25] Nang E.E., Gitau Ngunjiri S.A., Wu Y., Salim A., Tai E.S., Lee J., Van Dam R.M. (2011). Validity of the International Physical Activity Questionnaire and the Singapore Prospective Study Program physical activity questionnaire in a multiethnic urban Asian population. BMC Med. Res. Methodol..

[26] Ainsworth B.E., Haskell W.L., Whitt M.C., Irwin M.L., Swartz A.M., Strath S.J., O’Brien W.L., Bassett D.R., Schmitz K.H., Emplaincourt P.O. (2000). Compendium of physical activities: An update of activity codes and MET intensities. Med. Sci.Sports Exerc..

[27] Whitton C., Rebello S.A., Lee J., Tai E.S., van Dam R.M. (2018). A Healthy Asian A Posteriori Dietary Pattern Correlates with A Priori Dietary Patterns and Is Associated with Cardiovascular Disease Risk Factors in a Multiethnic Asian Population. J. Nutr..

[28] World Health Organization (2004). Appropriate body-mass index for Asian populations and its implications for policy and intervention strategies. Lancet (Lond. Engl.).

[29] World Health Organization (2011). Waist Circumference and Waist–hip Ratio: Report of a WHO Expert Consultation; Geneva, Switzerland, 8–11 December 2008.

[30] Leong S.L., Madden C., Gray A., Waters D., Horwath C. (2011). Faster Self-Reported Speed of Eating Is Related to Higher Body Mass Index in a Nationwide Survey of Middle-Aged Women. J. Am. Diet. Assoc..

[31] Nagahama S., Kurotani K., Pham N.M., Nanri A., Kuwahara K., Dan M., Nishiwaki Y., Mizoue T. (2014). Self-reported eating rate and metabolic syndrome in Japanese people: Cross-sectional study. BMJ Open.

[32] Ohkuma T., Hirakawa Y., Nakamura U., Kiyohara Y., Kitazono T., Ninomiya T. (2015). Association between eating rate and obesity: A systematic review and meta-analysis. Int. J. Obes..

[33] Robinson E., Almiron-Roig E., Rutters F., de Graaf C., Forde C.G., Tudur Smith C., Nolan S.J., Jebb S.A. (2014). A systematic review and meta-analysis examining the effect of eating rate on energy intake and hunger. Am. J. Clin. Nutr..

[34] Bolhuis D.P., Forde C.G., Cheng Y., Xu H., Martin N., de Graaf C. (2014). Slow Food: Sustained Impact of Harder Foods on the Reduction in Energy Intake over the Course of the Day. PLoS ONE.

[35] Forde C.G., van Kuijk N., Thaler T., de Graaf C., Martin N. (2013). Texture and savoury taste influences on food intake in a realistic hot lunch time meal. Appetite.

[36] McCrickerd K., Lim C.M., Leong C., Chia E.M., Forde C.G. (2017). Texture-Based Differences in Eating Rate Reduce the Impact of Increased Energy Density and Large Portions on Meal Size in Adults. J. Nutr..

[37] de Graaf C. (2012). Texture and satiation: The role of oro-sensory exposure time. Physiol. Behav..

[38] Morton G.J., Cummings D.E., Baskin D.G., Barsh G.S., Schwartz M.W. (2006). Central nervous system control of food intake and body weight. Nature.

[39] Rolls E.T. (2007). Sensory processing in the brain related to the control of food intake. Proc. Nutr. Soc..

[40] Kokkinos A., le Roux C.W., Alexiadou K., Tentolouris N., Vincent R.P., Kyriaki D., Perrea D., Ghatei M.A., Bloom S.R., Katsilambros N. (2010). Eating slowly increases the postprandial response of the anorexigenic gut hormones, peptide YY and glucagon-like peptide-1. J. Clin. Endocr. Metab..

[41] Li J., Zhang N., Hu L., Li Z., Li R., Li C., Wang S. (2011). Improvement in chewing activity reduces energy intake in one meal and modulates plasma gut hormone concentrations in obese and lean young Chinese men1–3. Am. J. Clin. Nutr..

[42] Zhu Y., Hsu W.H., Hollis J.H. (2013). Increasing the number of masticatory cycles is associated with reduced appetite and altered postprandial plasma concentrations of gut hormones, insulin and glucose. Br. J. Nutr..

[43] Lee K.S., Kim D.H., Jang J.S., Nam G.E., Shin Y.N., Bok A.R., Kim M.J., Cho K.H. (2013). Eating rate is associated with cardiometabolic risk factors in Korean adults. Nutr. Metab. Cardiovasc. Dis..

[44] Song L.L., Venkataraman K., Gluckman P., Chong Y.S., Chee M.W., Khoo C.M., Leow M.K., Lee Y.S., Tai E.S., Khoo E.Y. (2016). Smaller size of high metabolic rate organs explains lower resting energy expenditure in Asian-Indian Than Chinese men. Int. J. Obes. (Lond).

[45] Sun L., Ranawana D.V., Tan W.J.K., Quek Y.C.R., Henry C.J. (2015). The impact of eating methods on eating rate and glycemic response in healthy adults. Physiol. Behav..

[46] Ng R.Y.-X., Wong Y.-S., Yeo J.-Y., Koh C.L.-Z., Wilson C., Ken-En Gan S. (2018). The associations between dietary practices and dietary quality, biological health indicators, perceived stress, religiosity, culture, and gender in multicultural Singapore. J. Ethn. Foods.

[47] Wee M.S.M., Goh A.T., Stieger M., Forde C.G. (2018). Correlation of instrumental texture properties from textural profile analysis (TPA) with eating behaviours and macronutrient composition for a wide range of solid foods. Food Funct..

[48] Forde C.G., Mars M., DeGraaf K. (2020). Ultra-processing or Oral Processing? A role for Energy Density and Eating Rate in Moderating Energy Intake from Processed Foods. Nutr. Res. Rev..

[49] van den Boer J., Werts M., Siebelink E., de Graaf C., Mars M. (2017). The Availability of Slow and Fast Calories in the Dutch Diet: The Current Situation and Opportunities for Interventions. Foods.

[50] Mechanick J.I., Kushner R.F., Sugerman H.J., Gonzalez-Campoy J.M., Collazo-Clavell M.L., Spitz A.F., Apovian C.M., Livingston E.H., Brolin R., Sarwer D.B. (2009). American Association of Clinical Endocrinologists, The Obesity Society, and American Society for Metabolic & Bariatric Surgery medical guidelines for clinical practice for the perioperative nutritional, metabolic, and nonsurgical support of the bariatric surgery patient. Obes. (Silver Spring).

[51] Ford A.L., Bergh C., Södersten P., Sabin M.A., Hollinghurst S., Hunt L.P., Shield J.P.H. (2010). Treatment of childhood obesity by retraining eating behaviour: Randomised controlled trial. BMJ.

[52] Galhardo J., Hunt L.P., Lightman S.L., Sabin M.A., Bergh C., Sodersten P., Shield J.P. (2012). Normalizing eating behavior reduces body weight and improves gastrointestinal hormonal secretion in obese adolescents. J. Clin. Endoc. Metab..

[53] Hamilton-Shield J., Goodred J., Powell L., Thorn J., Banks J., Hollinghurst S., Montgomery A., Turner K., Sharp D. (2014). Changing eating behaviours to treat childhood obesity in the community using Mandolean: The Community Mandolean randomised controlled trial (ComMando)—A pilot study. Health Technol. Assess..

[54] Teo P.S., Forde C.G., Meiselman H.L. (2019). The Impact of Eating Rate on Energy Intake, Body Composition and Health. Handbook of Eating and Drinking: Interdisciplinary Perspectives.

